# Synergistic removal of *Staphylococcus aureus* biofilms by using a combination of phage *Kayvirus rodi* with the exopolysaccharide depolymerase Dpo7

**DOI:** 10.3389/fmicb.2024.1438022

**Published:** 2024-08-07

**Authors:** Ana Catarina Duarte, Lucía Fernández, Andrea Jurado, Ana Belén Campelo, Yang Shen, Ana Rodríguez, Pilar García

**Affiliations:** ^1^Instituto de Productos Lácteos de Asturias (IPLA-CSIC), Villaviciosa, Spain; ^2^DairySafe Group, Instituto de Investigación Sanitaria del Principado de Asturias (ISPA), Oviedo, Spain; ^3^Laboratory of Food Microbiology, Institute of Food, Nutrition and Health, Zurich, Switzerland

**Keywords:** *Staphylococcus aureus*, bacteriophage, exopolysaccharide depolymerase, biofilms, synergy

## Abstract

**Introduction:**

Bacteriophages have been shown to penetrate biofilms and replicate if they find suitable host cells. Therefore, these viruses appear to be a good option to tackle the biofilm problem and complement or even substitute more conventional antimicrobials. However, in order to successfully remove biofilms, in particular mature biofilms, phages may need to be administered along with other compounds. Phage-derived proteins, such as endolysins or depolymerases, offer a safer alternative to other compounds in the era of antibiotic resistance.

**Methods:**

This study examined the interactions between phage *Kayvirus rodi* with a polysaccharide depolymerase (Dpo7) from another phage (*Rockefellervirus IPLA7*) against biofilms formed by different *Staphylococcus aureus* strains, as determined by crystal violet staining, viable cell counts and microscopy analysis.

**Results and discussion:**

Our results demonstrated that there was synergy between the two antimicrobials, with a more significant decreased in biomass and viable cell number with the combination treatment compared to the phage and enzyme alone. This observation was confirmed by microscopy analysis, which also showed that polysaccharide depolymerase treatment reduced, but did not eliminate extracellular matrix polysaccharides. Activity assays on mutant strains did not identify teichoic acids or PNAG/PIA as the exclusive target of Dpo7, suggesting that may be both are degraded by this enzyme. Phage adsorption to *S. aureus* cells was not significantly altered by incubation with Dpo7, indicating that the mechanism of the observed synergistic interaction is likely through loosening of the biofilm structure. This would allow easier access of the phage particles to their host cells and facilitate infection progression within the bacterial population.

## 1 Introduction

The Gram-positive pathogen *Staphylococcus aureus* is currently recognized by the World Health Organization (WHO) as one of the top 10 global public health threats faced by humanity (FAO and WHO, 2019). Armed with an arsenal of virulence factors, this microbe can cause diverse infections ranging from mild to severe, including skin and soft tissue, osteoarticular, lung or device-related infections, endocarditis and potentially bacteremia (Tong et al., 2015). Indeed, *S. aureus* is a leading cause of hospital-acquired infections. On top of that, some strains of this pathogen secrete heat-stable enterotoxins that can resist thermal inactivation strategies and remain in foods (González-Martín et al., 2020). Because of these toxins, *S. aureus* is responsible for food poisoning outbreaks associated with the consumption of milk, cheeses, butter, and ham, among others (Ortega et al., 2010; Centers for Disease Control and Prevention, 2023). In the food environment, the main sources of contamination include workers and farm animals, with the latter also suffering often from staphylococcal infections (e.g., mastitis). Worryingly, the prevalence of multidrug-resistant isolates of *S. aureus* has been increasing, not only in the clinic but also along the food chain. The most concerning strains are those exhibiting methicillin and vancomycin resistance (MRSA and VRSA, respectively).

This microorganism is also capable of colonizing and forming biofilms on both biotic and abiotic surfaces. A biofilm is an agglomeration of bacteria attached to a surface embedded within an extracellular matrix that, in this pathogen, mainly consists of exopolysaccharide (PNAG/PIA), DNA and proteins (François et al., 2023). This structure provides the bacterial cells with protection from external factors such as antibiotics, disinfectants and host defense mechanisms (Costerton et al., 1999; de la Fuente-Núñez et al., 2013; Lister and Horswill, 2014). As a result, biofilms are much more difficult to eliminate than planktonic cells. In the case of *S. aureus*, the biofilm lifestyle is thought to be involved in most chronic infections, such as those related to indwelling medical devices, and in the persistence of this bacterium on food surfaces (Di Ciccio et al., 2015; Moormeier and Bayles, 2017).

In this scenario, phage therapy appears as a viable alternative to target this pathogen and, consequently, help to control the spread of antimicrobial resistance. Phages are viruses that infect bacteria and offer, as one of their main advantages, high host specificity, being innocuous for non-target microorganisms as well as for humans and animals (Principi et al., 2019). To prevent phage resistance development, phage therapy generally involves the administration of cocktails consisting of several phages that bind to different receptors on the bacterial surface (Labrie et al., 2010; Fernández et al., 2019). Another strategy is the simultaneous administration of phages together with other types of drugs. For example, several studies have demonstrated that the combination of bacteriophages with antibiotics sometimes increases the chances of a successful treatment (Diallo and Dublanchet, 2022; Roszak et al., 2022). This phenomenon is known as phage-antibiotic synergy (PAS). However, other researchers have found that antagonism between phages and antibiotics may also occur, making it necessary to study each individual interaction (Nicholls et al., 2023). More recently, a study reported that simultaneous treatment of staphylococcal biofilms with a phage and a phage-derived lytic enzyme displayed better efficacy than either antimicrobial alone (Duarte et al., 2021). This result highlights the possibilities offered by combining a phage with proteins derived from other bacterial viruses.

Phage-encoded polysaccharide depolymerases are enzymes that help the virus penetrate the carbohydrate barrier posed by the host cell envelope in order to access its receptor and inject the viral genome into the cell (Latka et al., 2017). These proteins are very diverse and their substrates include lipopolysaccharide (LPS), the capsule (CPS), polysaccharidic extracellular polymeric substance (EPS) or even wall teichoic acid (WTA) (Myers et al., 2015; Latka et al., 2017). These enzymes may be grouped into two classes, hydrolases and lyases, depending on how they degrade their target carbohydrate (Guo et al., 2023). In a previous work, Gutiérrez et al. (2015a) identified a pre-neck appendage protein (Dpo7) derived from Staphylococcus phage vB_SepiS-phiIPLA7, containing a pectate lyase domain. Dpo7 exhibits antibiofilm activity, but it does not kill biofilm cells; it just triggers their dispersion to the planktonic phase (Gutiérrez et al., 2015a). On the other hand, the virulent staphylophage *Kayvirus rodi* (phiIPLA-RODI) can readily kill biofilm cells, but it does not produce any matrix-degrading enzymes (Gutiérrez et al., 2015b). Within this context, the main goal of this work was to investigate the potential synergy between these two agents against biofilms formed by different *S. aureus* strains in an attempt to improve on the antibiofilm potential of phage phiIPLA-RODI.

## 2 Materials and methods

### 2.1 Bacterial strains, growth conditions, polysaccharide depolymerase and bacteriophages

The staphylococcal strains used in this study are included in Table 1. These strains were routinely grown at 37°C in TSB medium (tryptic soy broth, Scharlau Microbiology, Barcelona, Spain) with shaking, or on plates containing TSB supplemented with 1.5% (w/v) agar (Roko, S.A., Llanera, Asturias, Spain) (TSA).

**Table 1:** Staphylococcal strains used in this study.

Strain*	Description	Genome accession number*	Reference
Sa IPLA16	Meat industry surface	CP134617	(Gutiérrez et al., 2012)
Sa 15981	Clinical strain	NA	(Valle et al., 2003)
Sa V329	Cow mastitis	JAGTJH000000000	(Cucarella et al., 2001)
Sa BIM1	Phage resistant mutant	JAGTJI000000000	(Duarte et al., 2021)
Sa Newman	Clinical strain	NC_009641	(Duthie and Lorenz, 1952)
Sa JE2	MRSA, clinical strain	NZ_CP020619	(Fey et al., 2013)
Sa SA113	Derived from NCTC 8325	NZ_JASTSW000000000	(Iordanescu and Surdeanu, 1976)
SA113Δ*ica*	*ica* mutant	NA	(Cramton et al., 1999)
Se F12	Human mastitis	NA	(Delgado et al., 2009)

*Sa, *S. aureus*; Se, *Staphylococcus epidermidis*; NA, not available.

Recombinant protein expression was carried out using *Escherichia coli* BL21 carrying the gene coding for Dpo7 cloned into plasmid pET21a as described by Gutiérrez et al. (2020). *E. coli* was routinely grown in LB medium (Luria Bertani broth; Sigma-Aldrich, St. Louis, MO, United States), supplemented with 1 mM IPTG (GoldBio, St. Louis, MO, USA) and 100 μg/mL ampicillin (Sigma-Aldrich, St. Louis, MO, USA) when necessary. The bacteriophage-encoded polysaccharide depolymerase Dpo7 was subsequently purified as described previously (Gutiérrez et al., 2020). Visual analysis and quantification of the purified protein were performed by SDS-PAGE and the quick Start Bradford Protein Assay Kit (Bio-Rad Laboratories, USA), respectively.

Phage phiIPLA-RODI was routinely propagated on *S. aureus* IPLA16 using the double agar overlay technique (Labrie et al., 2010). Briefly, 100 μL of phage suspension (approximately 10^8^ PFUs) was added to TSA plates containing 100 μL of the host (approximately 10^7^ CFUs) and mixed with 5 mL of TSB top agar (TSB supplemented with 0.7% (w/v) agar). The plates were incubated overnight at 37°C after which 3 mL of TSB were added to each Petri dish. The plates were subsequently incubated at room temperature for 3 h, with gentle shaking. Lastly, the TSB and top agar with the eluted phages were collected and centrifuged (10,000 rpm, 4°C, 30 min). The recovered supernatant was filtered using cellulose acetate syringe filters with a pore size of 0.45 μm and 25 mm in diameter (VWR, Spain) and stored at 4°C until further use.

### 2.2 Dpo7 activity diffusion assay

For the diffusion test, overnight cultures of different strains were diluted 1:100 in 20 mL of TSB containing 1.2% agar and poured onto a Petri dish. These plates were allowed to dry and subsequently incubated for 24 h at 37°C. The next day, small holes were made with a micropipette tip in which we added 40 μL from a Dpo7 solution (approximately 10 μM). The protein elution buffer was used as a negative control. Results were analyzed after further incubation of the plates for 24 h at 37°C.

### 2.3 Biofilm formation and treatment

Overnight cultures of each strain were diluted 1:100 (v/v) in fresh TSB supplemented with 0.25% (v/v) glucose (Merck, Darmstadt, Germany) (TSBg), and 1 mL of each bacterial suspension was inoculated into a well of a 24-well polystyrene microplate (Thermo Scientific, NUNC, Madrid, Spain). These plates were incubated for 24 h at 37°C. After that, the planktonic phase was removed and the biofilms were washed twice with phosphate-buffered saline (PBS; 137 mM NaCl, 2.7 mM KCl, 10 mM Na_2_HPO_4_, 2 mM KH_2_PO_4_ [pH 7.4]). Biofilms were then treated with 0.5 mL of TSB medium alone, as a control, or medium supplemented with different concentrations of Dpo7 (1 and 2 μM) and/or phage phiIPLA-RODI (1 × 10^8^ PFU/ml and 1 × 10^9^ PFU/ml) and incubated for 24 h at 37°C. Again, the planktonic phase was removed, and the adhered cells were washed twice with PBS. The control provided information on the number of cells in the biofilm in the absence of antimicrobial treatment and allowed us to quantify the impact of the individual and combination treatments.

To determine the number of viable cells, the planktonic phase was removed and, after washing the biofilms twice with PBS, 1 mL of this same buffer was added to each well and biofilms were scraped with a pipette tip. Then, 10-μl droplets from tenfold serial dilutions of each cell suspension were placed onto TSA plates, incubated for 24 h at 37°C and the colony forming units in the biofilm per unit area of well surface (CFU/cm^2^) were determined. Interactions between the phage and Dpo7 were estimated according to the following equation based on Chaudhry et al. (2017):

Interaction index = [S(phage+Dpo7) - S(control)] - [[S(phage) - S(control)] + [S(Dpo7) - S(control)]].

In which S = log_10_ CFU/cm^2^ for a specific treatment.

Values of this index between −0.2 and 0.2 indicated an additive interaction, whereas antagonism and synergy were found when the value was < −0.2 or > 0.2, respectively.

The total biomass of biofilms was quantified by crystal violet staining. After removal of the planktonic phase, the wells were washed twice with PBS and then 1.5 mL of 0.1% (v/v) crystal violet was added to each well and incubated for 15 min at room temperature. The staining was then removed, and the wells were washed twice with water and allowed to dry at room temperature. The remaining dye was solubilized by adding 1.5 mL of 33% (v/v) acetic acid to each well and the absorbance at 595 nm (A_595_) was measured in a microplate reader (Benchmark Plus Microplate Spectrophotometer; Bio-Rad Laboratories, Hercules, CA, United States).

### 2.4 Confocal microscopy

Biofilms were formed on 2-well μ-slides with a glass bottom (ibidi, USA) by inoculating 2 mL from a cell suspension prepared as above and allowing growth for 24 h at 37°C. After removing the planktonic phase, wells were washed with PBS and cells were treated with TSB supplemented with phage phiIPLA-RODI at 10^9^ PFU/ml, 2 μM Dpo7 or a combination of both. Control wells were treated with TSB alone to assess biofilm development under the same conditions but in the absence of any antimicrobial treatment. Following 24 h of treatment, the planktonic phase was removed again. The adhered cells were washed with PBS and subsequently stained with Live/Dead® BacLightTM kit (Invitrogen AG, Basel, Switzerland) and WGA Alexa Fluor® 647 conjugate (Invitrogen, Eugene, Oregon, USA) following the manufacturers’ instructions. Briefly, 1 mL of PBS containing 1.5 μL of SYTO9, 1.5 μL of propidium iodide and 15 μL of WGA Alexa Fluor® 647 conjugate was added to each well. Samples were incubated at room temperature for 30 min and, following removal of the staining solution, observed with a confocal scanning laser microscope (DMi8, Leica Microsystems) using a 63× oil objective.

### 2.5 Extraction and purification of *Staphylococcus aureus* WTA polymers and monomers

WTA polymers from different *S. aureus* strains were extracted and purified as previously described with some modifications (Shen et al., 2017).

10 mL of overnight cultures of each *S. aureus* strain were inoculated in 1 L of TSB with 0.25% (v/v) glucose and incubated for 24 h at 37C. Then, bacterial cells were harvested by centrifugation at 7,000 × g for 10 min, resuspended in MilliQ water (15 mL per liter of culture), followed by heating at 100°C for 20 min and centrifuged at 7,000 × g for 10 min. The pellet was stored overnight at −20C.

Upon thawing, the cells were resuspended in 10 mL of Milli-Q water and then disrupted by two runs through a Pressure Cell Homogenizer (Stansted Fluid Power Ltd., Model SPCH-10, United Kingdom) at 200 MPa. After disruption, whole cells were centrifuged at 1,400 × g for 5 min and the supernatant was collected for WTA purification.

Since the cell wall material is in the supernatant, the suspension was spun down at 20,000 × g for 30 min. Afterwards the pellet was resuspended in 10 mL of SM buffer supplemented with magnesium (30 mM). Then, 10 μL of 1 U/mL DNase and 1 U/mL RNase were added and samples were incubated for 3 h at 37°C. Next, the same volume of 20 mg/mL proteinase K was added and incubation at 37°C continued for another 2 h. The cell walls were extracted by boiling the samples in the presence of 4% SDS. The insoluble fraction was recovered by centrifugation (20,000 × g, 30 min, 25°C). The pellet was washed with Milli-Q water and centrifuged again for 20 min at the same speed. This process was repeated five times.

Extraction of WTAs was carried out by resuspending the resulting pellet in 10 mL of 25 mM glycine/HCl buffer and heated in a water bath for 10 min at 100°C. Afterwards, samples were centrifuged for 30 min at 30,000 × g and the supernatant was transferred to a new tube. WTA extraction was repeated twice and the supernatants were mixed together. To finish, the supernatant was dialyzed against Milli-Q water and lyophilized for the next purification step.

Next, the lyophilized product was solved in Milli-Q water and WTAs were purified by anion exchange chromatography by using a HiTrap DEAE FF column. 10 mM Tris–HCl (pH 7.5) was chosen as buffer, with a gradient elution ranging from 0 to 1 M NaCl. The outflow was measured with a UV detector at the following wavelengths: 205 and 212 nm (both indicating carbohydrate signal) as well as 280 nm (for protein signal).

After sugar separation, fractions were collected, lyophilized and resuspended in 1 mL of PBS. Afterwards, the samples were analyzed by anion exchange chromatography and ultra-performance liquid chromatography tandem mass spectrometry (UPLC-MS/MS).

Purified WTA polymers were depolymerized into monomeric repeating units by hydrolysis of the phosphodiester bonds using 48% hydrofluoric acid for 20 h at 0°C. The WTA monomers were then lyophilized and subjected to UPLC-MS/MS analysis as previously described. All data were collected and processed using MassLynx software, version 4.1 (Waters Corp., United States), and MS spectra were background-corrected by subtracting the signals between 0 and 1 min of their respective chromatograms.

### 2.6 Phage adsorption assays

Biofilms were grown as described above but inoculating 2 mL into each well of a 12-well polystyrene microplate (Thermo Scientific, NUNC, Madrid, Spain). These plates were incubated for 24 h at 37°C. The following day, the planktonic phase was removed and the biofilms were washed twice with PBS. Then, the adhered cells were harvested in 1 mL of PBS and diluted down to an OD_600_ = 1. The resulting suspension was then divided into 1-ml aliquots. Dpo7 was added to one of the tubes at a concentration of 2 μM, while the same amount of buffer without enzyme was added to the untreated control. Both samples were incubated at 37°C for 4 h, after which cells were pelleted by centrifugation and resuspended in 900 μL of PBS. Next, 100 μL of phage suspension was added to each sample (final titer: 10^7^ PFU/ml) and PBS containing phage with no cells was used as a control. All samples were then incubated for 5 min at room temperature. After this time, the tubes were centrifuged for 3 min at 10,000 × g, and the adsorption rate was calculated according to the following equation:

$\mathrm{adsorption rate}=\left[\begin{array}{l} \left(\begin{array}{l} \mathrm{phage titer in supernatant of control}-\\ \mathrm{phage titer in supernatant sample} \end{array}\right)/\\ \left(\mathrm{phage titer in supernatant of control}\right) \end{array}\right]\;x\;100$

### 2.7 Statistical analysis

All experiments were carried out with at least three independent biological replicates. Statistical analysis was performed with Student’s t-test, using the Holm-Sidak method or Welch’s correction with GraphPad Prism 6 software. *p*-values lower than 0.05 were considered significant.

## 3 Results and discussion

### 3.1 Dpo7 exhibits activity against diverse *Staphylococcus aureus* strains

To establish the potential interactions between the phage-derived polysaccharide depolymerase Dpo7 and phage phiIPLA-RODI for biofilm removal, we chose several strains from diverse origins (food environment, clinic, cows with mastitis) with different biofilm-forming abilities and biofilm matrix composition. Both *S. aureus* V329 and *S. aureus* 15981 form strong biofilms, but the extracellular matrix of V329 is mostly composed of Bap protein and eDNA, while that of strain 15981 is rich in polysaccharides (Gutiérrez et al., 2014). On the other hand, strains IPLA16, JE2 and Newman are all poor biofilm formers (Jurado et al., 2024).

**Figure 1:**
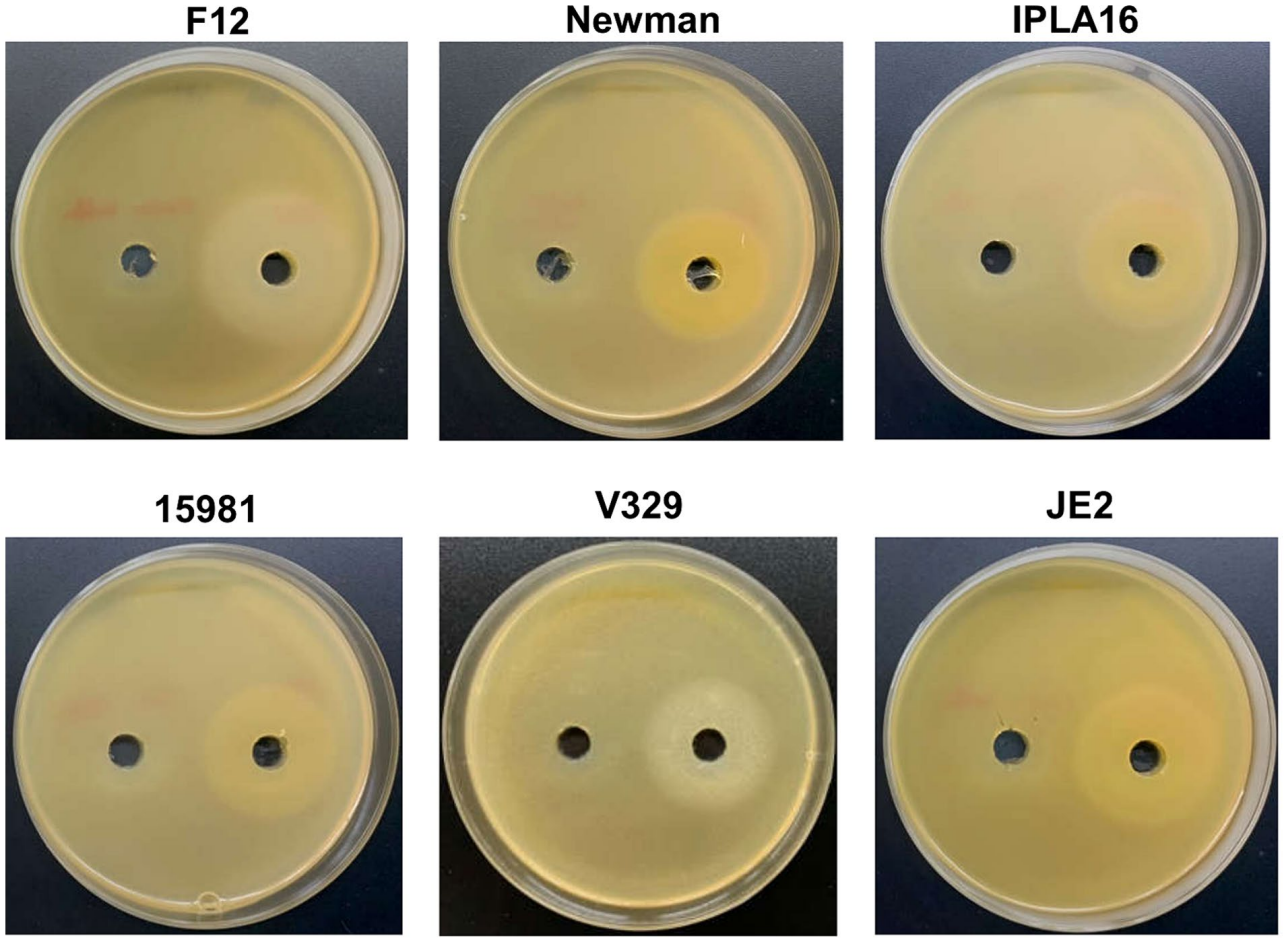
Activity of depolymerase Dpo7 on different staphylococcal strains (indicated on top of each photograph) as determined by the diffusion assay. The protein (40 μL from an 800 μg/mL stock) or buffer alone were, respectively, added to the right and left well. Activity was observed as a turbid halo around the well.

Before conducting the interaction assays, we examined the activity of depolymerase Dpo7 on the different *S. aureus* strains and one *S. epidermidis* strain, which was included as a positive control. According to previous data obtained by Gutiérrez et al. (2015a), strain V329 was expected to be a negative control of depolymerase activity. However, as can be seen in Figure 1, all the strains tested displayed a halo indicating Dpo7 enzymatic activity (left hole). No haloes were observed when buffer alone was present (right hole), demonstrating that the turbid zones were due to the protein. Based on these results, all five *S. aureus* strains (15981, V329, Newman, JE2 and IPLA16) were included in the biofilm treatment experiments.

### 3.2 Dpo7 and phage phiIPLA-RODI act synergistically to degrade *Staphylococcus aureus* biofilms

**Figure 2:**
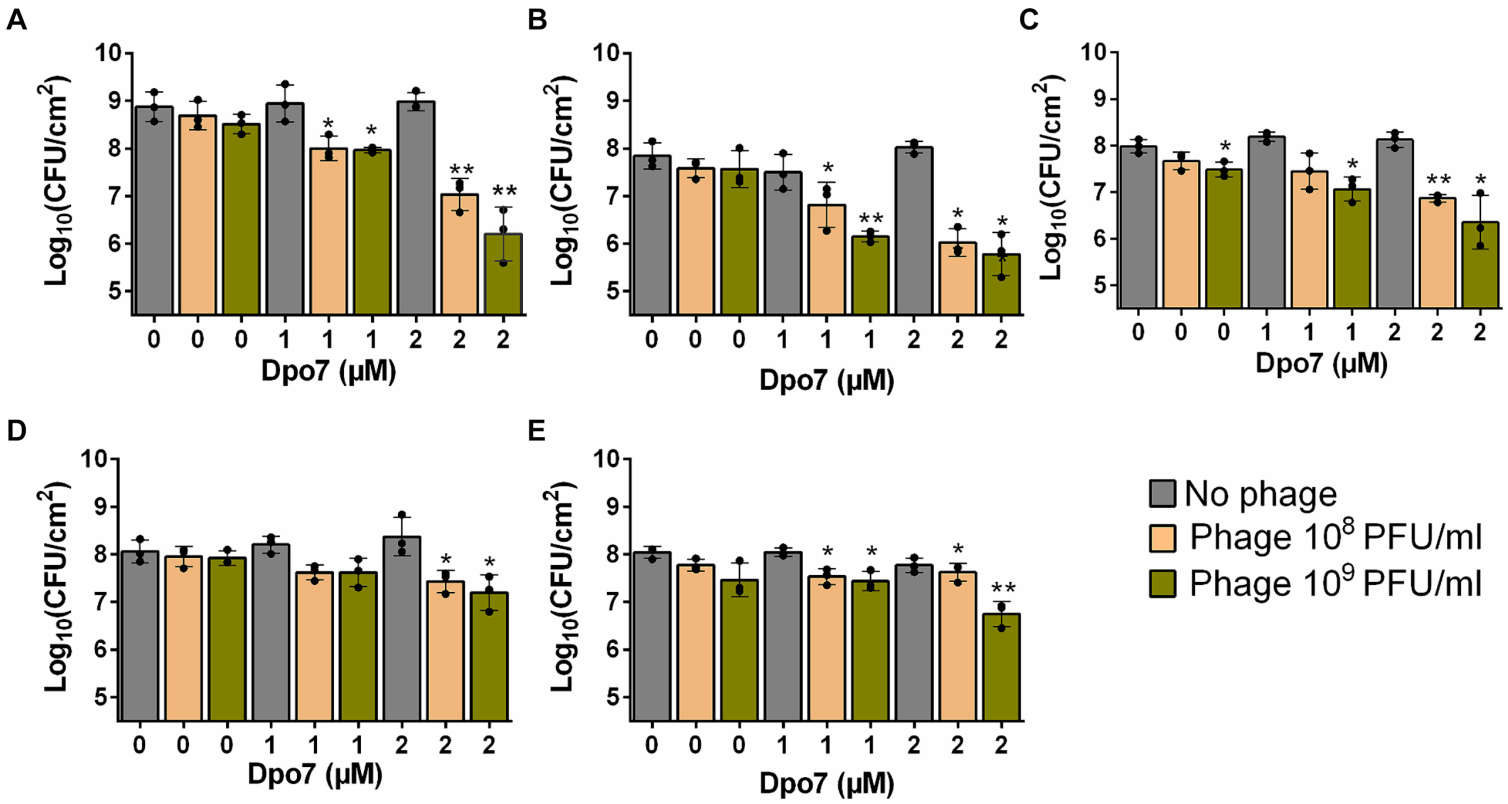
Treatment of *S. aureus* preformed biofilms with different combinations of bacteriophage phiIPLA-RODI and depolymerase Dpo7. 24-h biofilms formed by *S. aureus* 15981 **(A)**, V329 **(B)**, Newman **(C)**, JE2 **(D)** and IPLA16 **(E)** were treated with 10^8^ or 10^9^ PFU/ml of phage (orange and green bars, respectively), 1 or 2 μM of depolymerase, or a combination of both antimicrobials. Biofilms were treated for 24 h at 37°C. TSB medium alone was added to the control wells (gray bars). The graphs show the viable cell counts observed after each treatment. Data represent the means ± standard deviations of three independent experiments. Bars with an asterisk are statistically different from the untreated control. *p-value <0.05; **p-value <0.01; ***p-value <0.001; and ****p-value <0.0001.

Preformed biofilms of the aforementioned strains were treated with different combinations of phage and enzyme. The obtained results revealed a synergistic interaction between the two antimicrobials in most of the combinations tested (Table 2), with greater reductions in viable cell counts observed when phage and Dpo7 were applied together compared to the individual treatments (Figure 2). Indeed, treatment with phage or depolymerase alone did not significantly affect the number of viable cells compared to an untreated control, with the exception of strain Newman treated with 10^9^ PFU/ml of phage phiIPLA-RODI (Figure 2C). In contrast, most of the combinations tested led to significant reductions in viable cell counts.

**Table 2:** Interaction indices calculated for combinations of phiIPLA-RODI and Dpo7.

Strain	Treatment	Interaction index*
15981	10^8^ PFU/ml phage +1 μM Dpo7	**0.76**
	10^8^ PFU/ml phage +2 μM Dpo7	**1.77**
	10^9^ PFU/ml phage +1 μM Dpo7	**1.62**
	10^9^ PFU/ml phage +2 μM Dpo7	**2.42**
V329	10^8^ PFU/ml phage +1 μM Dpo7	**0.41**
	10^8^ PFU/ml phage +2 μM Dpo7	**1.80**
	10^9^ PFU/ml phage +1 μM Dpo7	**1.07**
	10^9^ PFU/ml phage +2 μM Dpo7	**2.02**
Newman	10^8^ PFU/ml phage +1 μM Dpo7	**0.42**
	10^8^ PFU/ml phage +2 μM Dpo7	**0.94**
	10^9^ PFU/ml phage +1 μM Dpo7	**0.63**
	10^9^ PFU/ml phage +2 μM Dpo7	**1.27**
JE2	10^8^ PFU/ml phage +1 μM Dpo7	**0.48**
	10^8^ PFU/ml phage +2 μM Dpo7	**0.83**
	10^9^ PFU/ml phage +1 μM Dpo7	**0.45**
	10^9^ PFU/ml phage +2 μM Dpo7	**1.04**
IPLA16	10^8^ PFU/ml phage +1 μM Dpo7	**0.24**
	10^8^ PFU/ml phage +2 μM Dpo7	−0.12
	10^9^ PFU/ml phage +1 μM Dpo7	0.03
	10^9^ PFU/ml phage +2 μM Dpo7	**0.44**

*Values > 0.2 and < −0.2 were considered synergistic (bold) and antagonistic, respectively. Values between − 0.2 and 0.2 were considered indicative of an additive interaction.

In the case of strain 15981, the number of biofilm cells decreased by 0.87 and 1.84 log units for Dpo7 concentrations of 1 μM and 2 μM, respectively, combined with 10^8^ PFU/ml of phage, and by 0.91 and 2.67 log units for depolymerase concentrations of 1 μM and 2 μM, respectively, combined with 10^9^ PFU/ml of phage (Figure 2A). The results were similar for strain V329 (Figure 2B). Thus, when combining phage at 10^8^ PFU/ml with protein at 1 μM and 2 μM, the reduction in viable cells was 1.02 and 1.82 log units, respectively. Similarly, the combination of Dpo7 with phiIPLA-RODI at 10^9^ PFU/ml resulted in a decrease of 1.69 and 2.06 log units for depolymerase concentrations of 1 μM and 2 μM, respectively. For strain Newman, the number of viable cells decreased by 0.54, 1.12, 0.92 and 1.63 log units when biofilms were treated with 10^8^ PFU/ml with 1 μM Dpo7, 10^8^ PFU/ml with 2 μM Dpo7, 10^9^ PFU/ml with 1 μM Dpo7 and 10^9^ PFU/ml with 2 μM Dpo7, respectively (Figure 2C). The same treatments led to reductions of 0.44, 0.63, 0.44 and 0.87 log units in the number of biofilm cells for *S. aureus* JE2 (Figure 2D). IPLA16 was the only strain tested for which synergy between the phage and the protein was the weakest according to the interaction index calculation (Table 2). Nonetheless, we observed that the combination of phiIPLA-RODI at 10^9^ PFU/ml with 2 μM Dpo7 led to a decrease in the number of viable cells of 1.29 log units. This value exceeded the impact of the sum of the individual treatments, since the phage and protein alone at the same concentrations resulted in average reductions of 0.58 and 0.27 log units, respectively, which add up to 0.85 in total. Similarly, treatment with 1 μM Dpo7 or 10^8^ PFU/ml of phage phiIPLA-RODI independently led to a reduction of 0 or 0.2 log units, while the combination decreased the number of viable cells by 0.5 log units.

This synergistic interaction with phage phiIPLA-RODI has a broader range than the mechanical antibiofilm activity reported by Gutiérrez et al. (2015a), which was limited to strains forming biofilms consisting mostly of exopolysaccharide (PNAG/PIA). Most studies assessing potential interactions between a purified phage-derived depolymerase and bacteriophages had found antagonism due to degradation of the phage receptor by the enzyme. This is for instance the case of *Klebsiella* depolymerase Dep_kpv74, which has capsule-degradation activity (Volozhantsev et al., 2022) and the depolymerase of *Acinetobacter* phages K38 (Domingues et al., 2021) and PMK14 (Abdelkader et al., 2022). In contrast, the depolymerase from *Klebsiella* phage KP34 helps phage KP15, probably by exposing its receptor on the bacterial surface (Latka and Drulis-Kawa, 2020). An even greater effect was observed when co-infecting with the two phages. Nevertheless, given the specificity of *Klebsiella* phages and the activity of the depolymerase on the main phage receptor, the capsule, this synergy seems likely to be fairly limited to certain strains.

### 3.3 Impact of the phiIPLA-RODI combination with Dpo7 on biofilm biomass and integrity

Once we established the impact of the phage-depolymerase combination on the number of viable biofilm cells, we studied its effect on total biomass and overall biofilm integrity on the two strong biofilm-forming strains, namely 15981 and V329.

**Figure 3:**
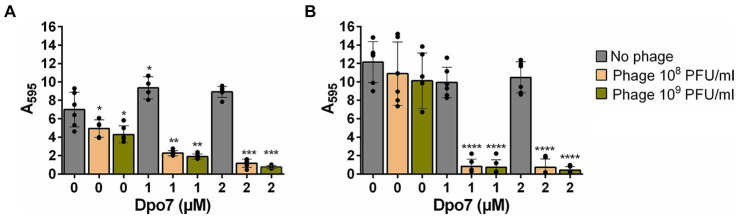
Impact of the treatment with different combinations of bacteriophage phiIPLA-RODI and depolymerase Dpo7 on *S. aureus* preformed biofilms. 24-h biofilms formed by *S. aureus* 15981 **(A)** and V329 **(B)** were treated with 10^8^ or 10^9^ PFU/ml of phage (orange and green bars, respectively), 1 or 2 μM of depolymerase Dpo7, or a combination of both antimicrobials for 24 h at 37°C. TSB medium alone was added to the control wells (gray bars). The graphs show the amount of total biomass as determined by crystal violet staining and subsequent measurement of absorbance at 595 nm. Data represent the means ± standard deviations of three independent experiments. Bars with an asterisk are statistically different from the untreated control. *p-value <0.05; **p-value <0.01; ***p-value <0.001; and ****p-value <0.0001.

Regarding the total biomass, there was a significant decrease for all the different combinations tested compared to the individual antimicrobials (Figure 3). In strain 15981, the phage alone significantly reduced the amount of attached biomass at both phage titers (by 29 and 39% for 10^8^ and 10^9^ PFU/ml, respectively) (Figure 3A). However, the reduction percentage in the mixed treatments varied between 68 and 89%. The difference was even more noticeable for strain V329, given that neither the phage nor the protein had any significant impact on total biomass when used separately. In contrast, the four combinations tested decreased the amount of total biomass between 93 and 97% (Figure 3B).

**Figure 4:**
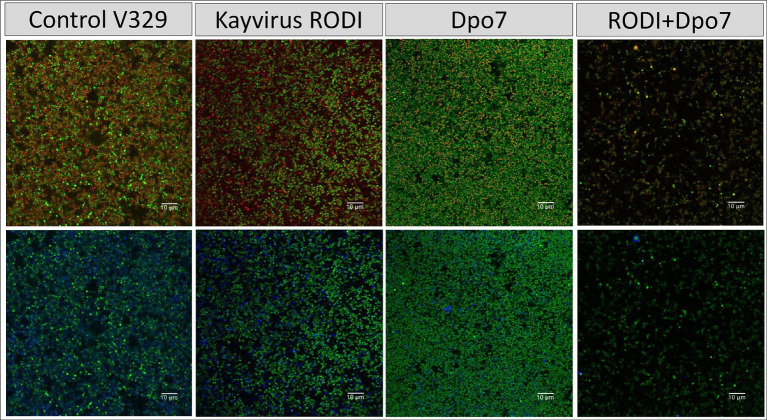
Treatment of biofilms formed by *S. aureus* V329 with phiIPLA-RODI (10^9^ PFU/ml), Dpo7 (2 μM), a combination of both or TSB alone (control). Biofilms were grown for 24 h at 37°C and subsequently treated for the same duration. After removal of the treatment, the attached cells were stained with SYTO® 9, propidium iodide and Wheat Germ Agglutinin (WGA) Alexa Fluor® 647 conjugate. Green represents live cells, red represents dead cells or eDNA and blue indicates the presence of polysaccharides with N-acetylglucosamine residues.

Next, we sought to compare more closely the microscopic structure of biofilms of these two strains that had been subjected to four different treatments, namely TSB alone (control), phiIPLA-RODI at 10^9^ PFU/ml, 2 μM Dpo7 and a phage-protein combination. In the case of *S. aureus* V329, the effect of the different treatments on the amount of viable cells mirrors the data presented in Figure 2B. Indeed, only the combined treatment led to a visible decrease in bacterial cells (Figure 4). Additionally, treatment with Dpo7 with or without phage resulted in a noticeable change in the amount of polysaccharide dyed with WGA Alexa Fluor® 647.

**Figure 5:**
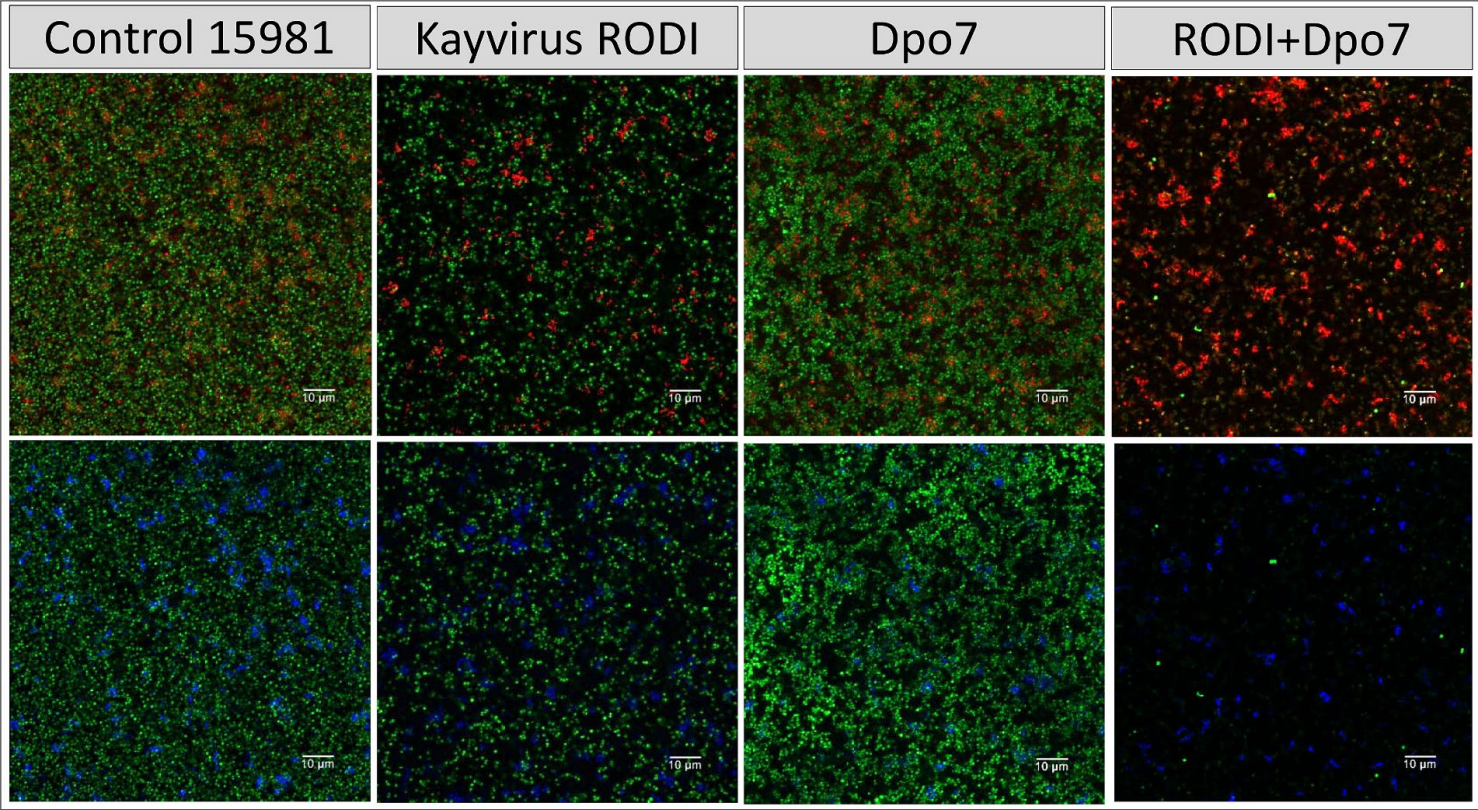
Treatment of biofilms formed by *S. aureus* 15981 with phiIPLA-RODI (10^9^ PFU/ml), Dpo7 (2 μM), a combination of both or TSB alone (control). Biofilms were grown for 24 h at 37°C and subsequently treated for the same duration. After removal of the treatment, the attached cells were stained with SYTO® 9, propidium iodide and Wheat Germ Agglutinin (WGA) Alexa Fluor® 647 conjugate. Green represents live cells, red represents dead cells or eDNA and blue indicates the presence of polysaccharides with N-acetylglucosamine residues.

The results observed for strain 15981 were very similar (Figure 5). However, the phage alone did have an impact on the number of biofilm cells in this strain, albeit not as dramatic as the combination treatment. Also, Dpo7 only reduced slightly the amount of stained polysaccharides, probably due to the larger amount of these compounds in this strain compared to V329.

### 3.4 Assessment of potential targets of depolymerase Dpo7 in *Staphylococcus aureus* strains

The inherent antibiofilm effect of depolymerase Dpo7 was limited to strains that form biofilms with a high content in EPS according to the results obtained by Gutiérrez et al. (2015a). The only strain tested here that really fits into that description is 15981. However, the synergy between this enzyme and phage phiIPLA-RODI is also observed for poor biofilm-forming strains (Newman, IPLA16 and JE2), as well as for strain V329, whose biofilms consist mainly of protein and eDNA (Gutiérrez et al., 2014). This prompted us to assess the potential of different extracellular or cell-wall polysaccharides to be Dpo7 degradation targets. To do that, we examined the presence of genes involved in polysaccharide production in the genomes of most strains included in this study, with the exception of strain 15981 whose genome is not available.

First, we searched for mutations affecting capsule production because it is considered to somewhat hinder phage infection in this pathogen (Wilkinson and Holmes, 1979). *S. aureus* Newman and *S. aureus* JE2 have been previously described to be capsule positive and negative, respectively. Regarding the other two strains, V329 appears to have an intact *cap* locus, while IPLA16 has a mutation in gene *capD* that would result in a non-functional product. Therefore, it does not seem likely that the capsule is the degradation target of Dpo7 that explains its promoting phage predation, given that some of our strains are capsule negative. Moreover, the microscopy analysis described above shows that depolymerase treatment has an effect on a polysaccharide to which wheat germ agglutinin can bind. As far as we know, the *S. aureus* capsule does not have such interactions.

Peptidoglycan does contain N-acetylglucosamine residues, and it has been shown to be agglutinated by WGA. However, it does not seem a probable Dpo7 target either since Gutiérrez et al. (2015a) already demonstrated that this enzyme does not exhibit lytic activity by carrying out turbidity reduction assays, which are commonly used to detect and quantify peptidoglycan hydrolase activity.

**Figure 6:**
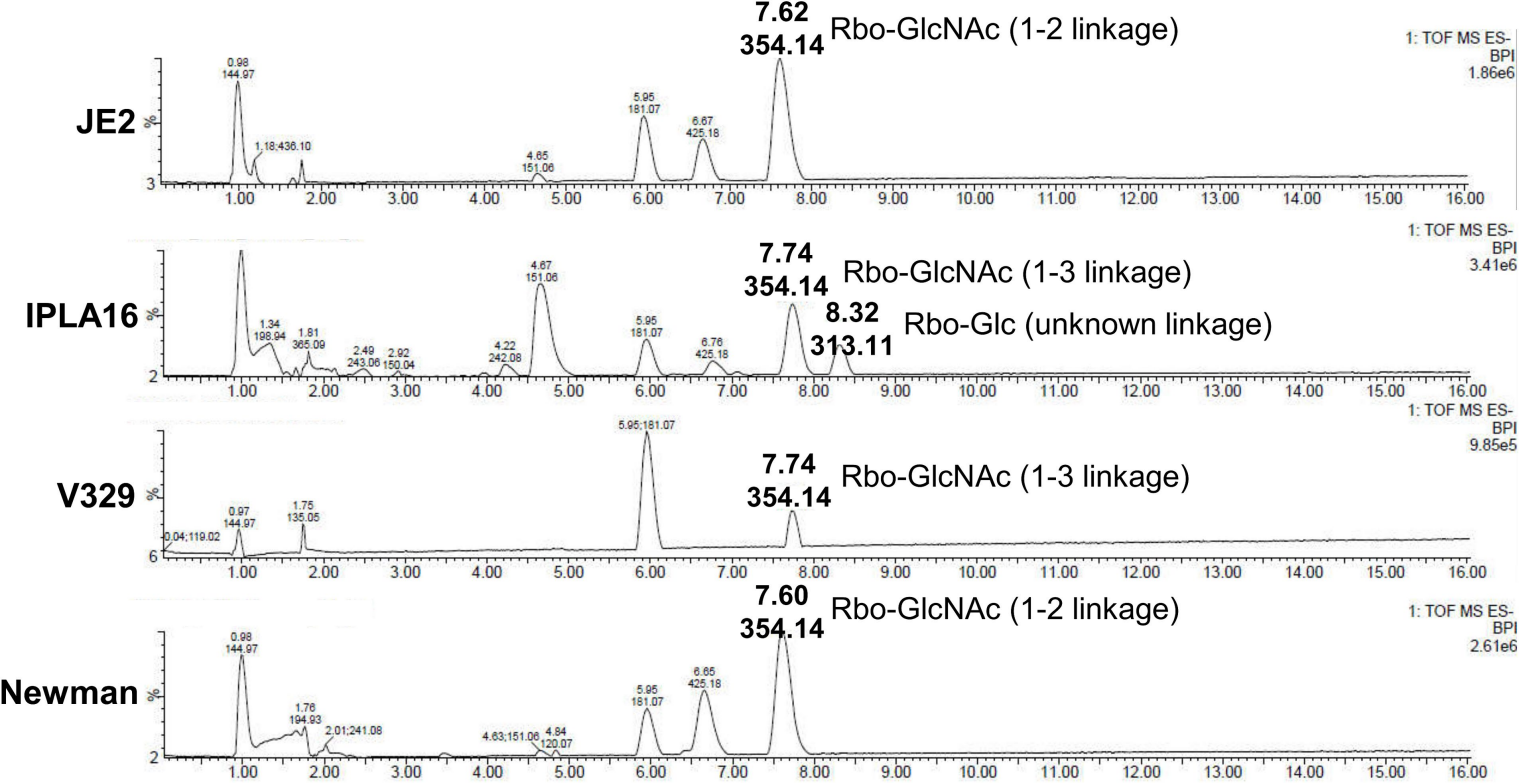
Structural analysis of *S. aureus* WTA repeating units by UPLC-MS. Liquid chromatographic separation and MS-based identification of carbohydrate residues within *S. aureus* WTAs. Peaks are labeled with their respective retention time [*Rt*; (min)] and base peak ion [M-H] (*m*/*z*).

**Figure 7:**
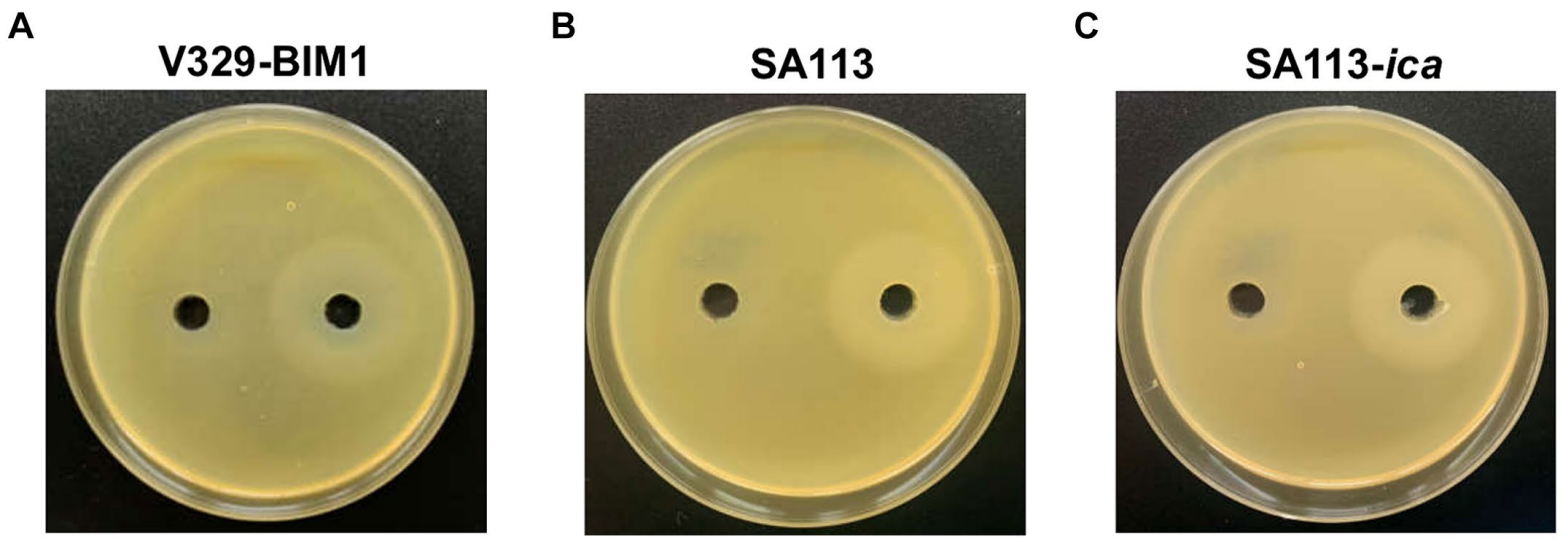
Impact of mutations in teichoic acid biosynthesis gene *tagO* and the *ica* operon on the activity of Dpo7 as determined by the diffusion assay. The protein (40 μL from an 800 μg/mL stock) or buffer alone were, respectively, added to the right and left well. Activity was observed as a turbid halo around the well. The strains used were V329-BIM1 **(A)**, SA113 **(B)** and SA113-ica **(C)**.

We also examined the genes involved in WTA production in these four strains and found that the genes responsible for biosynthesis of the WTA backbone as well as *tarS* were seemingly intact in all of them. There were differences, however, regarding gene *tarM*. More specifically, V329 and IPLA16 displayed mutations leading to the synthesis of truncated proteins, resulting in the lack of α-glycosylation in these strains. UPLC-MS analysis confirmed that all four strains produce WTA as well as the two different glycosylation patterns of V329 and IPLA16 compared to Newman and JE2 (Figure 6). WTA would therefore be a candidate target as it is present in at least four of the tested strains. Additionally, glycosylated WTA would bind WGA, so it might be one of the polysaccharides dyed with the Alexa Fluor 647 fluorophore that decreases after Dpo7 treatment (Lotan et al., 1975). To test this hypothesis, we carried out the plate diffusion assay with strain V329-BIM1, a V329-derived strain that has a mutation in *tagO*, the gene coding for the first protein involved in WTA biosynthesis (Duarte et al., 2021). However, as can be seen in Figure 7A, the halo indicating activity of depolymerase Dpo7 was observed as intensely as in the wild-type strain (Figure 1). Although these results do not completely discard WTA as a Dpo7 target, they do indicate, at the very least, that it is not the only one.

Finally, we looked at genes involved in PNAG/PIA biosynthesis, which mostly consists of N-acetylglucosamine residues and would therefore bind WGA. We did not find any mutation that would necessarily abrogate PNAG/PIA production in any of the strains. However, V329 is known to form biofilms whose matrix consists mostly of proteins and eDNA, and strains Newman, JE2 and IPLA16 are poor biofilms formers (Gutiérrez et al., 2014; Jurado et al., 2024). Even though none of these strains would be expected to produce a large amount of extracellular polysaccharide, they may still have a significant amount, whose degradation facilitates access of the phage to the target cells, explaining the synergy observed in this study. Therefore, exopolysaccharide could still be a target of Dpo7, as predicted by Gutiérrez et al. (2015a). We put this hypothesis to the test by carrying out the diffusion assay with strain SA113 and its isogenic mutation devoid of the entire *ica* operon. However, Dpo7 exhibited activity on both strains (Figures 7B,C). This does not necessarily mean that the enzyme cannot degrade PNAG/PIA, but it is clear that it is not the only target. Another potential target would be the newly identified surface polysaccharide Ssc (Lei et al., 2024). This molecule containing N-acetylgalactosamine was shown to affect phage susceptibility. In any case, further enzymatic experiments to assess degradation of the different potential target polysaccharides will be necessary to identify the specific targets of this protein.

### 3.5 Dpo7 treatment does not affect phage adsorption

With the aim of studying the synergy between Dpo7 and phiIPLA-RODI in greater detail, we explored the possibility that the activity of the depolymerase would have an effect on phage adsorption to biofilm cells of strain V329. However, our results indicated no significant difference in the percentage of phage particles that bind to the host cells in samples treated with Dpo7 compared to untreated samples (*p*-value = 0.34). Thus, the adsorption rates of untreated and Dpo7-treated samples were 64.79 ± 6.47% and 59.89 ± 9.35%, respectively. Similar results had been previously obtained with planktonic cells (data not shown). Therefore, it does not seem that this is the mechanism of the positive impact of enzymatic treatment on the killing ability of the phage. It seems more likely that, as stated above, exogenously added Dpo7 can loosen up the biofilm matrix, making bacterial cells more accessible to phiIPLA-RODI. In that sense, the results observed here are not that dissimilar in terms of mechanism to those reported by (Fernández et al., 2021a, 2021b) when using a DNAse-phage combination for biofilm treatment. Besides Dpo7, other phage-derived depolymerases have demonstrated antibiofilm activity such as those encoded by phages infecting *Proteus mirabilis*, *K. pneumoniae* and *Pseudomonas aeruginosa* (Mi et al., 2019; Rice et al., 2021; Li et al., 2022). It would be interesting to determine if these enzymes can also promote elimination of biofilms by other phages infecting these same species.

## 4 Conclusion

This study highlights the potential of using certain phage-derived depolymerases as enhancers in phage-based antibiofilm products. Thus far, this is only the second study that identified synergy between phages and this type of enzymes and the first that is likely to have a broad range of action against diverse strains. Given the recalcitrant nature of biofilms and the increase in antimicrobial resistance in pathogenic bacteria, the use of phages can be a valuable weapon both in the clinic and in the food industry. However, it is essential to design strategies that allow maximizing the efficacy of phages against their target species. In that sense, our findings show that addition of Dpo7 can significantly enhance killing of biofilm-embedded *S. aureus* cells by phage phiIPLA-RODI. Moreover, the synergistic interaction between the phage and this enzyme is observed against strains with different matrix composition and, very importantly, with varying degrees of phage susceptibility. It would be interesting to determine if this same effect can be demonstrated for other staphylococcal phages. If so, this would open the potential for the development of surface decontamination products containing Dpo7 together with a phage cocktail aimed at eliminating *S. aureus* from biofilms formed on food surfaces. Likewise, Dpo7 could be used in phage therapy for the treatment of biofilm-associated infections. The data available at the moment does not appear to limit the activity of Dpo7 on one single polysaccharide, being PNAG/PIA and WTAs the most probable targets. Nonetheless, subsequent efforts should examine the enzymatic degradation of these molecules by Dpo7. The mechanism behind this synergy remains elusive, as improved phage adsorption could not be demonstrated after Dpo7 treatment of cells recovered from biofilms. However, we can speculate that there might be an improvement in the adsorption of phiIPLA-RODI within the complex structure of an intact biofilm. Another possibility is that Dpo7 degradation of surface polysaccharides facilitates injection of the phage genome into the host cell. Understanding the specific activity and the impact of this enzyme on its molecular targets will help use this depolymerase in a more precise manner. In any case, incorporation of Dpo7 in phage-based antibiofilm products against *S. aureus* seems like a promising strategy to optimize the antimicrobial impact of these viruses.

## Data availability statement

The raw data supporting the conclusions of this article will be made available by the authors, without undue reservation.

## Author contributions

AD: Writing – original draft, Formal analysis, Investigation, Visualization. LF: Writing – original draft, Conceptualization, Funding acquisition, Supervision. AJ: Investigation, Writing – review & editing. AC: Investigation, Writing – review & editing. YS: Conceptualization, Supervision, Writing – review & editing. AR: Funding acquisition, Supervision, Writing – review & editing. PG: Conceptualization, Funding acquisition, Supervision, Writing – review & editing.

## References

[ref1] Abdelkader K. Gutiérrez D. Latka A. Boeckaerts D. Drulis-Kawa Z. Criel B.. (2022). The specific capsule depolymerase of phage PMK34 sensitizes *Acinetobacter baumannii* to serum killing. Antibiotics (Basel) 11:677. doi: 10.3390/antibiotics11050677 35625321 PMC9137491

[ref2] Centers for Disease Control and Prevention. (2023). *Staphylococcal (staph) food poisoning*. Available at: https://www.cdc.gov/foodsafety/diseases/staphylococcal.html.

[ref3] Chaudhry W. N. Concepción-Acevedo J. Park T. Andleeb S. Bull J. J. Levin B. R. (2017). Synergy and order effects of antibiotics and phages in killing *Pseudomonas aeruginosa* biofilms. PLoS One 12:e0168615. doi: 10.1371/journal.pone.0168615, PMID: 28076361 PMC5226664

[ref4] Costerton J. W. Stewart P. S. Greenberg E. P. (1999). Bacterial biofilms: a common cause of persistent infections. Science 284, 1318–1322. doi: 10.1126/science.284.5418.1318 10334980

[ref5] Cramton S. E. Gerke C. Schnell N. F. Nichols W. W. Götz F. (1999). The intercellular adhesion (Ica) locus is present in Staphylococcus aureus and is required for biofilm formation. Infect. Immun. 67, 5427–5433. doi: 10.1128/IAI.67.10.5427-5433.1999 10496925 PMC96900

[ref6] Cucarella C. Solano C. Valle J. Amorena B. Lasa I. Penadés J. R. (2001). Bap, a *Staphylococcus aureus* surface protein involved in biofilm formation. J. Bacteriol. 183, 2888–2896. doi: 10.1128/JB.183.9.2888-2896.2001, PMID: 11292810 PMC99507

[ref7] De la Fuente-Núñez C. Reffuveille F. Fernández L. Hancock R. E. W. (2013). Bacterial biofilm development as a multicellular adaptation: antibiotic resistance and new therapeutic strategies. Curr. Opin. Microbiol. 16, 580–589. doi: 10.1016/j.mib.2013.06.013, PMID: 23880136

[ref8] Delgado S. Arroyo R. Jiménez E. Marín M. L. del Campo R. Fernández L.. (2009). *Staphylococcus epidermidis* strains isolated from breast milk of women suffering infectious mastitis: potential virulence traits and resistance to antibiotics. BMC Microbiol. 9:82. doi: 10.1186/1471-2180-9-82, PMID: 19422689 PMC2685400

[ref9] Di Ciccio P. Vergara A. Festino A. R. Paludi D. Zanardi E. Ghidini S.. (2015). Biofilm formation by *Staphylococcus aureus* on food contact surfaces: relationship with temperature and cell surface hydrophobicity. Food Control 50, 930–936. doi: 10.1016/j.foodcont.2014.10.048

[ref10] Diallo K. Dublanchet A. (2022). Benefits of combined phage–antibiotic therapy for the control of antibiotic-resistant bacteria: a literature review. Antibiotics 11, 1–11. doi: 10.3390/antibiotics11070839 PMC9311689 35884092

[ref11] Domingues R. Barbosa A. Santos S. B. Pires D. P. Save J. Resch G.. (2021). Unpuzzling friunavirus-host interactions one piece at a time: phage recognizes *Acinetobacter pittii* via a new K38 capsule depolymerase. Antibiotics (Basel) 10:1304. doi: 10.3390/antibiotics10111304, PMID: 34827242 PMC8614642

[ref12] Duarte A. C. Fernández L. de Maesschalck V. Gutiérrez D. Campelo A. B. Briers Y.. (2021). Synergistic action of phage phiIPLA-RODI and lytic protein CHAPSH3b: a combination strategy to target *Staphylococcus aureus* biofilms. NPJ Biofilms Microbiomes 7, 1–10. doi: 10.1038/s41522-021-00208-5 33888725 PMC8062563

[ref13] Duthie E. S. Lorenz L. L. (1952). Staphylococcal coagulase: mode of action and antigenicity. J. Gen. Microbiol. 6, 95–107. doi: 10.1099/00221287-6-1-2-95 14927856

[ref14] FAO and WHO. (2019). *Food safety, everyone’s business*. Available at: https://www.fao.org/3/ca4449en/ca4449en.pdf.

[ref15] Fernández L. Duarte A. C. Martínez B. Rodríguez A. García P. (2021a). Draft genome sequences of the bap-producing strain *Staphylococcus aureus* V329 and its derived phage-resistant mutant BIM-1. Am. Soc. Microbiol. 10, 20–22. doi: 10.1128/MRA.00500-21 PMC8281075 34264112

[ref16] Fernández L. Gutiérrez D. García P. Rodríguez A. (2019). The perfect bacteriophage for therapeutic applications—a quick guide. Antibiotics 8:126. doi: 10.3390/antibiotics8030126, PMID: 31443585 PMC6783975

[ref17] Fernández L. Gutiérrez D. García P. Rodríguez A. (2021b). Environmental pH is a key modulator of *Staphylococcus aureus* biofilm development under predation by the virulent phage phiIPLA-RODI. ISME J. 15, 245–259. doi: 10.1038/s41396-020-00778-w, PMID: 32963343 PMC7852692

[ref18] Fey P. D. Endres J. L. Yajjala V. K. Widhelm T. J. Boissy R. J. Bose J. L.. (2013). A genetic resource for rapid and comprehensive phenotype screening of nonessential *Staphylococcus aureus* genes. MBio 4, e00537–e00512. doi: 10.1128/mBio.00537-12, PMID: 23404398 PMC3573662

[ref19] François P. Schrenzel J. Götz F. (2023). Biology and regulation of staphylococcal biofilm. Int. J. Mol. Sci. 24:5218. doi: 10.3390/ijms24065218, PMID: 36982293 PMC10049468

[ref20] González-Martín M. Corbera J. A. Suárez-Bonnet A. Tejedor-Junco M. T. (2020). Virulence factors in coagulase-positive staphylococci of veterinary interest other than *Staphylococcus aureus*. Vet. Q. 40, 118–131. doi: 10.1080/01652176.2020.1748253 32223696 PMC7178840

[ref21] Guo Z. Liu M. Zhang D. (2023). Potential of phage depolymerase for the treatment of bacterial biofilms. Virulence 14:2273567. doi: 10.1080/21505594.2023.2273567, PMID: 37872768 PMC10621286

[ref22] Gutiérrez D. Briers Y. Rodríguez-Rubio L. Martínez B. Rodríguez A. Lavigne R.. (2015a). Role of the pre-neck appendage protein (Dpo7) from phage vB_SepiS-phiIPLA7 as an anti-biofilm agent in staphylococcal species. Front. Microbiol. 6:1315. doi: 10.3389/fmicb.2015.01315 26635776 PMC4658415

[ref23] Gutiérrez D. Delgado S. Vázquez-Sánchez D. Martínez B. Cabo M. L. Rodríguez A.. (2012). Incidence of Staphylococcus aureus and analysis of associated bacterial communities on food industry surfaces. Appl. Environ. Microbiol. 78, 8547–8554. doi: 10.1128/AEM.02045-12, PMID: 23023749 PMC3502933

[ref24] Gutiérrez D. Garrido V. Fernández L. Portilla S. Rodríguez A. Grilló M. J.. (2020). Phage lytic protein LysRODI prevents staphylococcal mastitis in mice. Front. Microbiol. 11:7. doi: 10.3389/fmicb.2020.00007, PMID: 32038593 PMC6989612

[ref25] Gutiérrez D. Ruas-Madiedo P. Martínez B. Rodríguez A. García P. (2014). Effective removal of staphylococcal biofilms by the endolysin LysH5. PLoS One 9:e107307. doi: 10.1371/journal.pone.0107307, PMID: 25203125 PMC4159335

[ref26] Gutiérrez D. Vandenheuvel D. Martínez B. Rodríguez A. Lavigne R. García P. (2015b). Two phages, phiIPLA-RODI and phiIPLA-C1C, lyse mono-and dual-species staphylococcal biofilms. Appl. Environ. Microbiol. 81, 3336–3348. doi: 10.1128/AEM.03560-14, PMID: 25746992 PMC4407228

[ref27] Iordanescu S. Surdeanu M. (1976). Two restriction and modification systems in *Staphylococcus aureus* NCTC 8325. J. Gen. Microbiol. 96, 277–281. doi: 10.1099/00221287-96-2-277, PMID: 136497

[ref28] Jurado A. Fernández L. Rodríguez A. García P. (2024). Prevalence of virulence-and antibiotic resistance-associated genotypes and phenotypes in *Staphylococcus aureus* strains from the food sector compared to clinical and cow mastitis isolates. Front. Cell. Infect. Microbiol. 14:1327131. doi: 10.3389/fcimb.2024.1327131, PMID: 38348375 PMC10859521

[ref29] Labrie S. J. Samson J. E. Moineau S. (2010). Bacteriophage resistance mechanisms. Nat. Rev. Microbiol. 8, 317–327. doi: 10.1038/nrmicro2315 20348932

[ref30] Latka A. Drulis-Kawa Z. (2020). Advantages and limitations of microtiter biofilm assays in the model of antibiofilm activity of Klebsiella phage KP34 and its depolymerase. Sci. Rep. 10:20338. doi: 10.1038/s41598-020-77198-5, PMID: 33230270 PMC7683578

[ref31] Latka A. Maciejewska B. Majkowska-Skrobek G. Briers Y. Drulis-Kawa Z. (2017). Bacteriophage-encoded virion-associated enzymes to overcome the carbohydrate barriers during the infection process. Appl. Microbiol. Biotechnol. 101, 3103–3119. doi: 10.1007/s00253-017-8224-6, PMID: 28337580 PMC5380687

[ref32] Lei M. G. Jorgenson M. A. Robbs E. J. Black I. M. Archer-Hartmann S. Shalygin S.. (2024). Characterization of Ssc, an N-acetylgalactosamine-containing *Staphylococcus aureus* surface polysaccharide. J. Bacteriol. 206:e0004824. doi: 10.1128/jb.00048-24, PMID: 38712944 PMC11112989

[ref33] Li P. Ma W. Shen J. Zhou X. (2022). Characterization of novel bacteriophage vB_KpnP_ZX1 and its depolymerases with therapeutic potential for K57 *Klebsiella pneumoniae* infection. Pharmaceutics 14:1916. doi: 10.3390/pharmaceutics14091916, PMID: 36145665 PMC9505181

[ref34] Lister J. L. Horswill A. R. (2014). *Staphylococcus aureus* biofilms: recent developments in biofilm dispersal. Front. Cell. Infect. Microbiol. 4, 1–9. doi: 10.3389/fcimb.2014.00178 25566513 PMC4275032

[ref35] Lotan R. Sharon N. Mirelman D. (1975). Interaction of wheat-germ agglutinin with bacterial cells and cell-wall polymers. Eur. J. Biochem. 55, 257–262. doi: 10.1111/j.1432-1033.1975.tb02158.x 809273

[ref36] Mi L. Liu Y. Wang C. He T. Gao S. Xing S.. (2019). Identification of a lytic *Pseudomonas aeruginosa* phage depolymerase and its anti-biofilm effect and bactericidal contribution to serum. Virus Genes 55, 394–405. doi: 10.1007/s11262-019-01660-4 30937696

[ref37] Moormeier D. E. Bayles K. W. (2017). *Staphylococcus aureus* biofilm: a complex developmental organism. Mol. Microbiol. 104, 365–376. doi: 10.1111/mmi.13634, PMID: 28142193 PMC5397344

[ref38] Myers C. L. Ireland R. G. Garrett T. A. Brown E. D. (2015). Characterization of wall teichoic acid degradation by the bacteriophage ϕ29 appendage protein GP12 using synthetic substrate analogs. J. Biol. Chem. 290, 19133–19145. doi: 10.1074/jbc.M115.662866, PMID: 26085106 PMC4521036

[ref39] Nicholls P. Clark J. R. Gu Liu C. Terwilliger A. Maresso A. W. (2023). Class-driven synergy and antagonism between a *Pseudomonas* phage and antibiotics. Infect. Immun. 91:e0006523. doi: 10.1128/iai.00065-23, PMID: 37404162 PMC10429645

[ref40] Ortega E. Abriouel H. Lucas R. Gálvez A. (2010). Multiple roles of *Staphylococcus aureus* enterotoxins: pathogenicity, superantigenic activity, and correlation to antibiotic resistance. Toxins (Basel) 2, 2117–2131. doi: 10.3390/toxins2082117, PMID: 22069676 PMC3153285

[ref41] Principi N. Silvestri E. Esposito S. (2019). Advantages and limitations of bacteriophages for the treatment of bacterial infections. Front. Pharmacol. 10, 1–9. doi: 10.3389/fphar.2019.00513 31139086 PMC6517696

[ref42] Rice C. J. Kelly S. A. O’Brien S. C. Melaugh E. M. Ganacias J. C. B. Chai Z. H.. (2021). Novel phage-derived depolymerase with activity against *Proteus mirabilis* biofilms. Microorganisms 9:2172. doi: 10.3390/microorganisms9102172, PMID: 34683494 PMC8539402

[ref43] Roszak M. Dołęgowska B. Cecerska-Heryć E. Serwin N. Jabłońska J. Grygorcewicz B. (2022). Bacteriophage-ciprofloxacin combination effectiveness depends on *Staphylococcus aureus*-*Candida albicans* dual-species communities’ growth model. Microb. Drug Resist. 28, 613–622. doi: 10.1089/mdr.2021.0324, PMID: 35404123

[ref44] Shen Y. Boulos S. Sumrall E. Gerber B. Julian-Rodero A. Eugster M. R.. (2017). Structural and functional diversity in Listeria cell wall teichoic acids. J. Biol. Chem. 292, 17832–17844. doi: 10.1074/jbc.M117.813964, PMID: 28912268 PMC5663882

[ref45] Tong S. Y. Davis J. S. Eichenberger E. Holland T. L. Fowler V. G. (2015). *Staphylococcus aureus* infections: epidemiology, pathophysiology, clinical manifestations, and management. Clin. Microbiol. Rev. 28, 603–661. doi: 10.1128/CMR.00134-14, PMID: 26016486 PMC4451395

[ref46] Valle J. Toledo-Arana A. Berasain C. Ghigo J. M. Amorena B. Penadés J. R.. (2003). SarA and not σB is essential for biofilm development by Staphylococcus aureus. Mol. Microbiol. 48, 1075–1087. doi: 10.1046/j.1365-2958.2003.03493.x 12753197

[ref47] Volozhantsev N. V. Borzilov A. I. Shpirt A. M. Krasilnikova V. M. Verevkin V. V. Denisenko E. A.. (2022). Comparison of the therapeutic potential of bacteriophage KpV74 and phage-derived depolymerase (ß-glucosidase) against *Klebsiella pneumoniae* capsular type K2. Virus Res. 322:198951. doi: 10.1016/j.virusres.2022.198951 36191686

[ref48] Wilkinson B. J. Holmes K. M. (1979). *Staphylococcus aureus* cell surface: capsule as a barrier to bacteriophage adsorption. Infect. Immun. 23, 549–552. doi: 10.1128/iai.23.2.549-552.1979, PMID: 154475 PMC414199

